# Epithelial to Mesenchymal Transition (EMT) in a Laryngeal Squamous Cell Carcinoma of a Horse: Future Perspectives

**DOI:** 10.3390/ani10122318

**Published:** 2020-12-07

**Authors:** Federico Armando, Francesco Godizzi, Elisabetta Razzuoli, Fabio Leonardi, Mario Angelone, Attilio Corradi, Daniela Meloni, Luca Ferrari, Benedetta Passeri

**Affiliations:** 1 Pathology Unit, Department of Veterinary Science, University of Parma, Strada del Taglio 10, 43126 Parma, Italy; federico.armando@unipr.it (F.A.); attilio.corradi@unipr.it (A.C.); luca.ferrari@unipr.it (L.F.); benedetta.passeri@unipr.it (B.P.); 2 Department of Veterinary Science (DIMEVET), University of Milan, Via dell‘Università 6, 26900 Lodi, Italy; francesco.godizzi@unimi.it; 3 National Reference Center of Veterinary and Comparative Oncology (CEROVEC), Piazza Borgo Pila 39/24, 16129 Genoa, Italy; daniela.meloni@izsto.it; 4 Department of Veterinary Science, Strada del Taglio 10, 43126 Parma, Italy; fabio.leonardi@unipr.it (F.L.); marioangelonevet@libero.it (M.A.)

**Keywords:** horse, squamous cell carcinoma of the larynx (SCCL), epithelial to mesenchymal transition (EMT), equine papillomavirus type 2 (EcPV2)

## Abstract

**Simple Summary:**

Squamous cell carcinoma (SCC) is one of the most common cancers in horses, and it can arise at any site on the skin and mucosae. Recent studies associated equine papillomavirus type 2 (EcPV2) infections with this type of cancers of the oral tract and genitals. Larynx and pharynx are frequently recognized as sites of SCC. In humans, squamous cell carcinoma of the larynx (SCCL) is a common cancer associated with papilloma virus (PV) infection and epithelial to mesenchymal transition (EMT). EMT can occur under different biological conditions, upon the same programmed changes: embryogenesis and organ development fibrosis, wound healing, and cancer metastases. This work reports for the first time in a SCCL of a horse a wide immunohistochemical EMT characterization, by analyzing main epithelial markers (E-cadherin, β-catenin, and pan-cytokeratin AE3/AE1), main mesenchymal markers (N-cadherin and vimentin), and the main EMT-related transcription factors (TWIST-1, ZEB-1, and HIF-1α). This work illustrates an example of tumor cell adaptation during the metastatic process in the equine SCCL, taking also into consideration the potential influence provided by EcPV2 oncoproteins on the EMT process.

**Abstract:**

Squamous cell carcinoma (SCC) is one of the most frequent tumors of skin and muco-cutaneous junctions in the horse. Equine papillomavirus type 2 (EcPV2) has been detected in equine SCC of the oral tract and genitals, and recently also in the larynx. As human squamous cell carcinoma of the larynx (SCCL), it is strongly etiologically associated with high-risk papillomavirus (h-HPV) infection. This study focuses on tumor cells behavior in a naturally occurring tumor that can undergo the so-called epithelial to mesenchymal transition (EMT). A SCCL in a horse was investigated by immunohistochemistry using antibodies against E-cadherin, pan-cytokeratin AE3/AE1, β-catenin, N-cadherin, vimentin, ZEB-1, TWIST, and HIF-1α. EcPV2 DNA detection and expression of oncogenes in SCC were investigated. A cadherin switch and an intermediate filaments rearrangement within primary site tumor cells together with the expression of the EMT-related transcription factors TWIST-1, ZEB-1, and HIF-1α were observed. DNA obtained from the tumor showed EcPV2 positivity, with E2 gene disruption and E6 gene dysregulation. The results suggest that equine SCCL might be a valuable model for studying EMT and the potential interactions between EcPV2 oncoproteins and the EMT process in SCCL.

## 1. Introduction

In horses and other equids, squamous cell carcinoma (SCC) is the most common malignant skin neoplasia, and accounts for 7–37% of equine skin lesions [1]. SCC can arise at any site on the skin and mucosa. However, non-pigmented skin and muco-cutaneous junctions have been reported to be preferential SCC sites [2,3]. Larynx and pharynx are reported to be sites of SCC development in horses. Equine oral SCC is invasive and lymph node metastases were reported in 30% of horses with oral SCC [4]. The longest survival time reported in 11 horses with laryngeal SCC was 4 months [4]. Squamous cell carcinoma of the larynx (SCCL) is the sixth most common human cancer and has strong etiologic association with smoking [5] and also with high-risk human papillomavirus (h-HPV) infection [6]. Indeed, about 26.6% of patients with SCCL are h-HPV positive. Particularly, h-HPV16 is the most common type (19.8%). Interestingly, equine papillomavirus type 2 (EcPV2) also has been recently associated with SCC of the penis, vulva, and stomach [7,8,9,10], as well as it has been recently detected in equine SCCL [11]. These data suggest that the horse is a good spontaneous animal model for studying the pathogenic mechanisms of this tumor in humans.

The transition of epithelial cells to a mesenchymal phenotype, the so-called epithelial to mesenchymal transition (EMT), can occur under different biological conditions, following the same programmed changes: embryogenesis and organ development [12], fibrosis [13], wound healing [14], and cancer metastases [15]. This process is characterized by decreased expression of the typical epithelial proteins (i.e., E-cadherin and cytokeratin) and, due to a prompt activation of the so-called “master genes regulators” (i.e., TWIST, ZEB, SNAIL1, SLUG), a mesenchymal phenotype is gradually acquired [16]. Epithelial cells loosen cell-to-cell adhesion structures, modulate their polarity, and rearrange their cytoskeleton, with intermediate filaments typically switching from cytokeratins to vimentin [17]. Moreover, in cells undergoing EMT, a cadherin switch takes place and is characterized by decreased E-cadherin and transiently increased N-cadherin expression [18,19,20]. Extensive studies focused on this event and proposed to use it for diagnostic, prognostic, and even therapeutic approaches [21]. To the authors’ knowledge, the current work reports, for the first time, a wide immunohistochemical EMT characterization in a SCCL of a horse, by analyzing main epithelial markers (E-cadherin, β-catenin, and cytokeratin), main mesenchymal markers (N-cadherin and vimentin), and the main EMT-related transcription factors (TWIST-1, ZEB-1, and HIF-1α). Therefore, naturally occurring tumors in domestic and farm animals represent a unique opportunity to study cancer in vivo [14]. The authors present these results from an equine SCCL in order to: (1) provide preliminary results for setting future studies on the equine species as suitable model to study EMT in human cancers, not only for penile carcinomas [14], but also for other mucosal carcinoma such as oral carcinoma, thus opening new perspectives for future studies in this field; (2) provide hints for future studies in horses regarding the potential interaction between EcPV oncoproteins and the EMT process.

## 2. Materials and Methods

### 2.1. Post-Mortem Analysis

The horse was euthanized due to poor clinical conditions and necropsy was immediately performed and followed by proper tissue sampling for histopathological evaluation.

### 2.2. Histolopathological Evaluation

Collected organ specimens were formalin-fixed (10% *v*/*v*, pH 7.4) and paraffin-embedded (Bio-Plast 56–58 °C, Bio-Optica, Milano, Italy). Samples were routinely processed, and 5-µm thick sections were stained using Mayer’s hematoxylin and eosin (HE).

### 2.3. Immunohistochemistry

The primary antibodies were titrated according to the manufacturer’s recommendations. Among the antibodies employed in this study, only vimentin and TWIST-1 are reported by the manufacturer as validated against equine tissues. The other antibodies are validated against either canine (E-cadherin, β-catenin, ZEB-1, HIF-1α, vimentin, and TWIST-1) or bovine (N-cadherin, pan-cytokeratin AE1/AE3) tissues. Therefore, different tissues (i.e., digestive tract, heart, kidney, and skin) were sampled from another horse which was unrelated to the study and did not present significant histopathological findings. In addition, heart and skin were sampled from a cow without significant histopathological findings, and digestive tract, kidney, and skin from a dog without significant histopathological findings. The canine and bovine tissues were examined together with the equine tissues, in order to obtain a preliminary evaluation of the antibody cross-reactivity on the equine species. Only those primary antibodies exhibiting cross-reactivity with the expected target tissue from the control canine or bovine tissues, compared to the equine tissues, were employed against the horse neoplastic tissue, namely the antibodies against E-cadherin, β-catenin, ZEB-1, HIF-1α, vimentin, TWIST-1, N-cadherin, and pan-cytokeratin AE1/AE3. Once that cross-reactivity was confirmed, the tumor samples from the horse were always examined together with the appropriate controls (canine or bovine tissues and the equine counterpart). Immunohistochemistry was performed as previously described [22] with minor modifications. Briefly, after dewaxing–rehydration, tissue sections were exposed to antigen retrieval; then, sections were cooled at room temperature for 20 min before being soaked into 3% H_2_O_2_ for 12 min. Slides were rinsed twice in PBS, pH 7.4, followed by serum blocking with normal goat or rabbit serum. Incubation with primary antibody was carried out overnight at 4 °C. After being washed twice in PBS, pH 7.4, the slides were incubated for 30 min with a biotinylated goat anti-rabbit, or a goat anti-mouse, or a rabbit anti-goat IgG antibody. Afterwards, an avidin–biotin complex (ABC) peroxidase kit (Vectastain, Elite, ABC-Kit PK-6100, Vector Labs, Burlingame, CA, USA) and 3′3′-diaminobenzidine (DAB) system (DAB-Kit-SK4100, Vector Labs) were used for the detection of antigen–antibody reactions. Nuclei were counterstained with Mayer’s hematoxylin. For negative controls, the primary antibodies were replaced by rabbit or goat serum, or Balb/c ascitic fluid at corresponding concentrations. Antibody details and positive controls are reported in Table 1. EMT markers expression was qualitatively analyzed in each whole section.

### 2.4. Detection of EcPV2 and Evaluation of Oncogene Expression

DNA and RNA extraction was performed to check EcPV2 presence and expression of oncogenes in SCCL. One sample of kidney obtained from a healthy horse was used as negative control. To prevent cross-contamination, a different blade was used for each sample. Four sections (5 µm) were obtained from FFPE samples for total acid nucleic extraction, which was performed using an AllPrep DNA FFPE Kit (Qiagen, Hilden, Germany) and an AllPrep RNA FFPE Kit (Qiagen, Hilden) in accordance with the manufacturer’s instructions. 

During extraction, samples were treated with DNase (RNase-Free DNase set, Qiagen). To evaluate DNA amplifiability, β-actin gene amplification was performed [7]; then, EcPV2-L1 DNA presence was tested using a Real-Time protocol previously described [7]. Briefly, TaqMan^®^ probe-based real-time PCR (CustomProbe 2× qPCR Master Mix, Canvax, Cordoba, Spain, cat. E0339) was performed using a CFX96™ Real-Time System (Bio-Rad, Rome, Italy). The reaction mix had a final concentration of 1× TaqMan^®^ master mix, 200 nM probe, 100 nM each primer combination, and 200 ng of DNA. Reverse transcription (RT) was performed using a SuperScript™ IV VILO™ Master Mix with ezDNase™ Enzyme (Invitrogen, ThermoFisher Scientific (Waltham, MA, USA), cat. 11766050) according to the manufacturer’s instructions; cDNA was used to check gene expression by TaqMan^®^ probe-based real-time PCR (CustomProbe 2× qPCR Master Mix, Canvax, Spain, cat. E0339) performed using a CFX96™ Real-Time System (Bio-Rad, Paris, France). The PCR mix (25 μL) had a final concentration of 1× TaqMan^®^ master mix, 200 nM probe, 100 nM each primer combination, and 5 μL of template (cDNA or DNA and RNA used as negative control to exclude possible contamination by genomic DNA). The thermal profile used for amplification was the following: 95 °C for 10 min, followed by 40 cycles at 95 °C for 15 s, and 60 °C for 60 s for annealing/extension and detection of the fluorescence signal. The fluorescence threshold limit was set automatically. Moreover, the expression of E2, E6, and L1 genes was evaluated by RT-real-time PCR using primers and probes (Table 2) and a protocol described by Porcellato and co-workers [7]. Briefly, RT was performed using a SuperScript™ IV VILO™ Master Mix (Invitrogen, ThermoFisher Scientific) according to the manufacturer’s instructions. The cDNA was used to check gene expression: 5 μL of template were added to 20 μL of PCR mix at a final concentration of 1× master mix (iTaq Universal Probes Supermix, Bio-Rad, Italy), 200 nM probe, and 100 nM each primer combination. RNA was used as control to exclude possible contamination by EcPV2 genomic DNA. Nuclease-free water was used as negative control. Each sample was tested in triplicate.

## 3. Results

### 3.1. Post-Mortem and Microscopical Analysis

**Figure 1:**
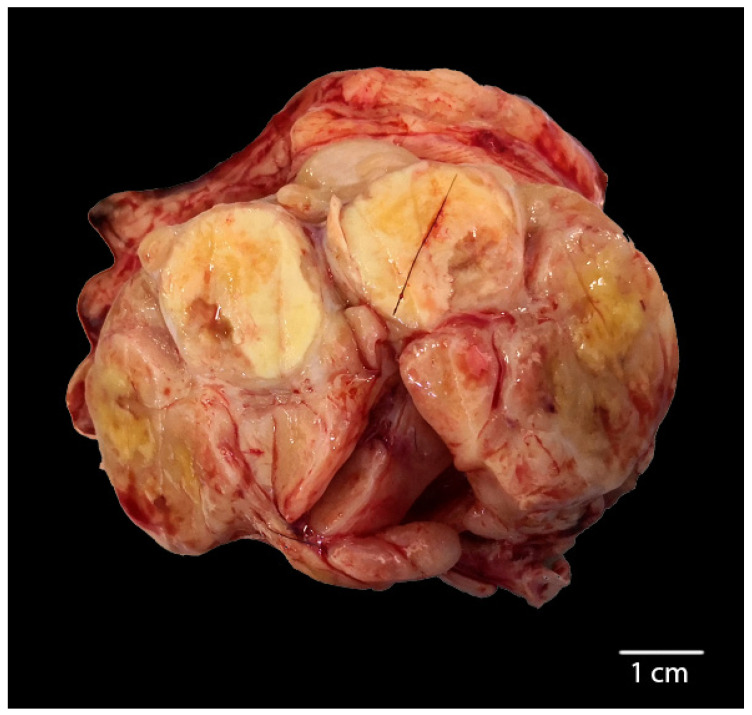
Cutting surface from the laryngeal region of the horse tumor sampled during the post-mortem analysis. A white to yellowish multilobular mass, located at the basis of the larynx, is partially ulcerated and shows multifocal central necrotic areas.

**Figure 2:**
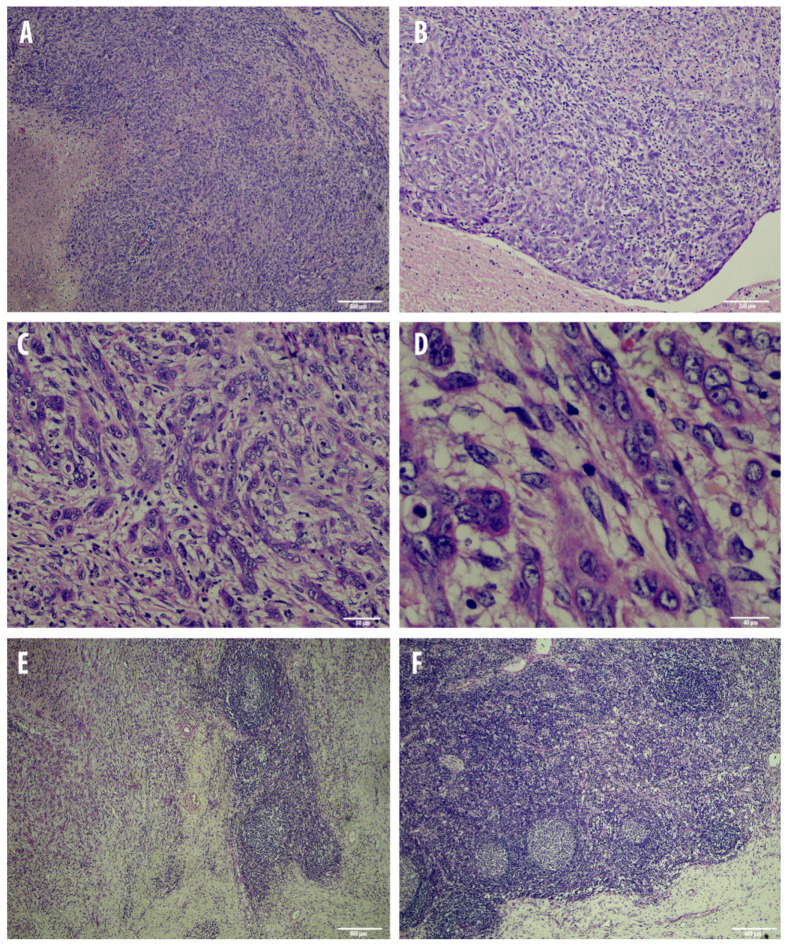
Overview of the histological findings of the squamous cell carcinoma of the larynx (**A**–**D**) and lymph node metastasis (**E**,**F**). (**A**): Neoplastic cells embedded in a moderate amount of fibrous collagen stroma are completely effacing the submucosa; of note, the multifocal necrotic areas within the tumor (4×, haematoxilyn–eosin, H&E). (**B**): neoplastic cells are variably arranged in anastomosing bands and chords occasionally forming lobules (10×, haematoxilyn–eosin, H&E). (**C**): Arborizing chords of variably differentiated squamous cells filling the submucosa and invading the nearby tissue (20×, haematoxilyn–eosin, H&E). (**D**): highly pleomorphic cell population with elongated, spindled cells often present in the invasive front of the tumor (40×, haematoxilyn–eosin, H&E). (**E**): few hyperplastic lymphoid follicles on the right with numerous invasive metastatic cells on the left (4×, haematoxilyn–eosin, H&E). (**F**): normal activated lymph node architecture scattered within the metastatic organ (4×, haematoxilyn–eosin, H&E).

A 17-year-old female, 550 Kg, Maremmano Horse was referred with severe clinical signs due to a dorso-cranial dislocation of the epiglottis which caused the reduction of 70% of the laryngeal lumen, diagnosed by an endoscopic exam, performed under sedation with butorphanol combined with detomidine [23]. The animal was humanely euthanized due to the poor condition. Necropsy revealed a locally expansive, multilobular, white-yellowish, firm mass localized at the base of the larynx. The lesion was partially ulcerated, with irregular margins and central necrotic areas (Figure 1). The neoplasia expanded to the nearby tissues and regional lymph nodes were markedly enlarged. Microscopically, the laryngeal tumor was completely effacing the submucosa, non-encapsulated, densely cellular, and poorly demarcated. Neoplastic cells were variably arranged in anastomosing bands and chords occasionally forming lobules embedded in a moderate amount of fibrous collagen stroma. Neoplastic cells were large, variably from polygonal to spindle-shaped with indistinct cell borders and an intermediate to high nuclear/cytoplasmic ratio (Figure 2). The cytoplasm was moderate and eosinophilic nuclei were large, round to oval, with vesicular chromatin and 1 or 2 round, basophilic nucleoli. Anisocytosis and anisokaryosis were high, and mitoses ranged from 0–2 per HPF (400×). Multifocally, there were wide areas of coagulative necrosis within the tumor. A moderate amount of small mature lymphocytes and plasma cells were found within and surrounding the tumor area. Remarkably, scattered tumor cells displayed a more prominent spindled shape. Interestingly, the regional lymph node was 80% effaced by necrotic and metastatic events (Figure 2). Numerous neoplastic cells with a morphology and histological pattern similar to those in the laryngeal tumor were found among resident lymphoid cells. Given this aggressive behavior of the neoplasia and this particular phenotypical change in morphology of the neoplastic cells which were variably from polygonal to elongated, an immunohistochemical panel for the EMT phenomenon was performed.

### 3.2. Cadherin Switch and Intermediate Filaments Rearrangements Suggest an EMT Phenomenon in Equine Laryngeal Squamous Cell Carcinomas

**Figure 3:**
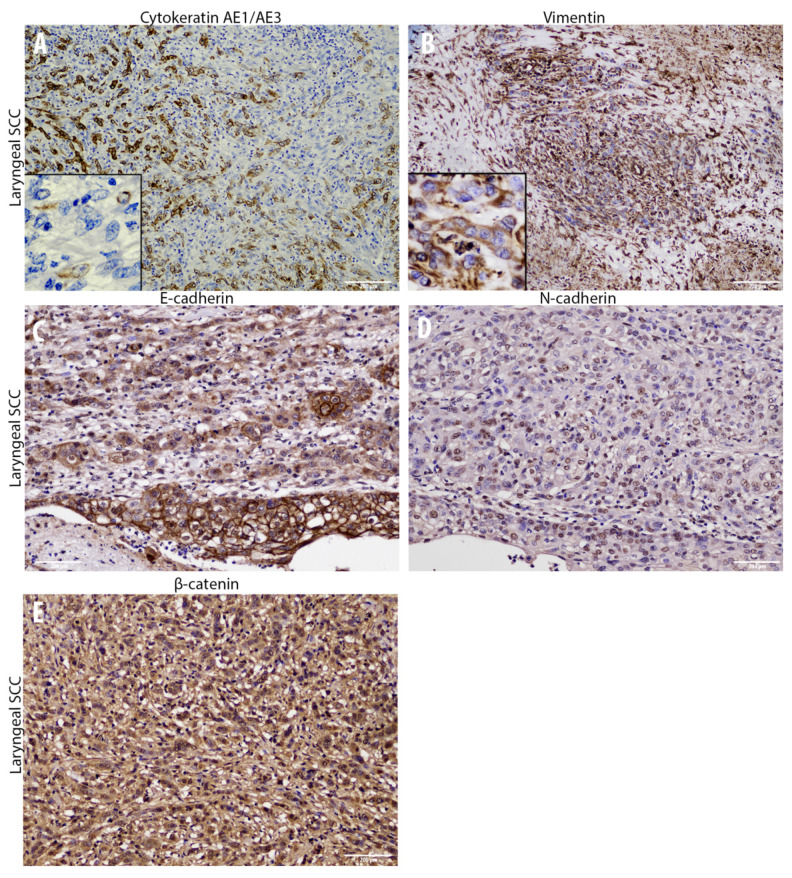
Overview of the immunohistochemical characterization of structural and adhesion molecules (**A**–**E**) of the laryngeal squamous cell carcinoma that supports an Epithelial to Mesenchymal transition (EMT) within the tumor. (**A**) immunohistochemistry for pan-cytokeratin AE1/AE3 displays a gradual cytoplasmic loss (insert) of this epithelial marker within neoplastic cells (10×). (**B**): Immunohistochemistry for vimentin reveals multifocal neoplastic cells characterized by elongated shape with a diffuse cytoplasmic expression (insert) of this mesenchymal marker (10×). (**C**): Immunohistochemistry displays a severe loss of membranous E-cadherin expression comparing the normal/dysplastic epithelium with the tumor cells invading the underlying tissue (10×). (**D**): A high number of nuclear N-cadherin immunolabeled neoplastic cells are present within the tumor (10×). (**E**): Immunohistochemistry reveals a moderate number of β-catenin cytoplasmic immunolabeled neoplastic cells together with numerous neoplastic cells also showing nuclear immunolabeling (10×).

**Figure 4:**
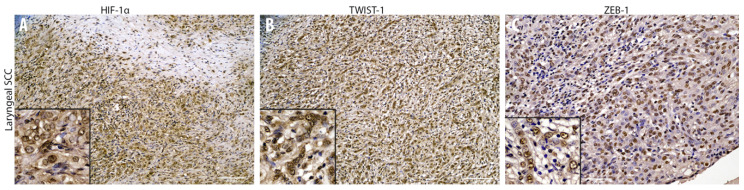
Overview of the immunohistochemical characterization of transcription factors (**A**–**C**) of the laryngeal squamous cell carcinoma that supports an Epithelial to Mesenchymal Transition (EMT) within the tumor. Immunohistochemical analysis reveals numerous nuclear immunolabeled neoplastic cells expressing HIF-1α (**A**, insert, 10×), TWIST-1 (**B**, insert, 10×), and ZEB-1 (**C**, insert, 10×) within the laryngeal squamous cell carcinoma.

**Figure 5:**
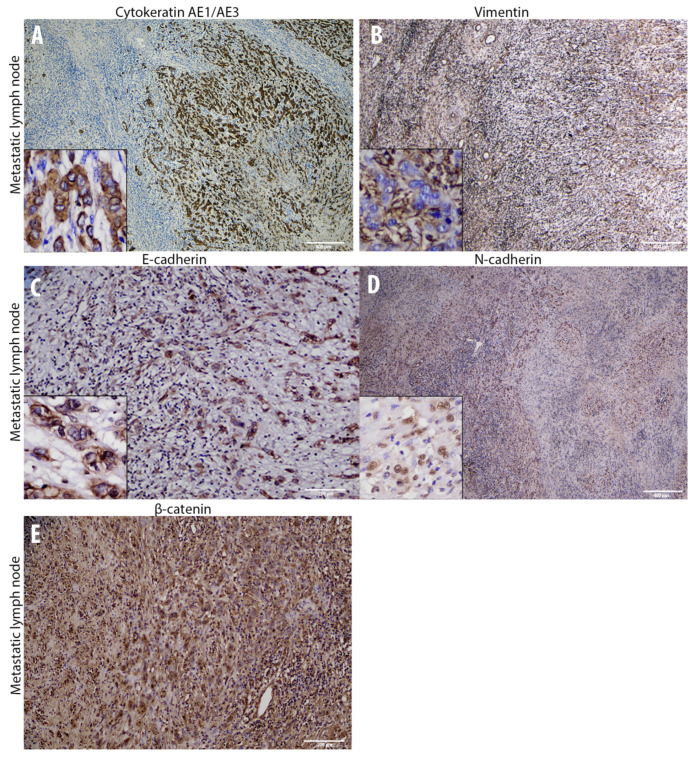
Overview of the immunohistochemical characterization of structural and adhesion molecules (**A**–**E**) of the metastatic lymph node that supports a Mesenchymal to Epithelial Transition (MET) within the tumor cells. (**A)** immunohistochemistry for pan-cytokeratin AE1/AE3 within the metastatic lymph node reveals a diffuse cytoplasmic (insert) immunolabeling in numerous metastatic neoplastic cells surrounded by resident lymphoid cells (4×). (**B**): Immunohistochemistry for vimentin reveals no positive immunolabeled metastatic neoplastic cells (insert) surrounded by positive stromal resident cells (4×). (**C**): Immunohistochemistry displays a gradual re-acquisition of membranous E-cadherin expression (insert) in metastatic neoplastic cells (10×). (**D**): A moderate number of nuclear N-cadherin immunolabeled neoplastic cells are present in the metastatic lymph node (4×). (**E**): Immunohistochemistry reveals a moderate number of β-catenin immunolabeled metastatic neoplastic cells showing either a cytoplasmic or a nuclear expression (10×).

**Figure 6:**
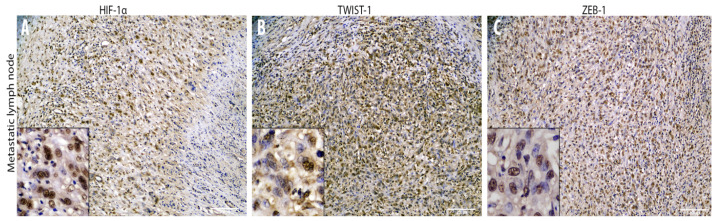
Overview of the immunohistochemical characterization of transcription factors (**A**–**C**) of the metastatic lymph node that supports a Mesenchymal to Epithelial Transition (MET) within the tumor cells. Immunohistochemical analysis reveals a moderate number of nuclearly immunolabeled metastatic neoplastic cells expressing HIF-1α (**A**, insert, 10×), TWIST-1 (**B**, insert, 10×), and ZEB-1 (**C**, insert, 10×) within the metastatic lymph node.

Immunohistochemical analysis of primary site tumor cells revealed an increased number of cells with cytoplasmic E-cadherin expression rather than membranous, together with a gradual overall loss of cells expressing this adhesion molecule moving towards the invasive front of the tumor (Figure 3). Interestingly, the number of cells expressing N-cadherin resulted to be increased within primary site tumor cells, even though it was expressed at nuclear level instead of being membranous (Figure 3). Interestingly, moving from the tumor center towards the invasive front, a decreased number of cells expressing cytokeratin was observed, while scattered neoplastic cells with a prominent mesenchymal morpholgy acquired cytoplasmic vimentin expression (Figure 3). Noteworthy, numerous neoplastic cells were scattered throughout the tumor area and expressed the EMT-related transcription factors TWIST-1, ZEB-1, and HIF-1α (Figure 4). In addition, β-catenin was found to be frequently expressed at a nuclear level, or (less frequently) cytoplasmically, rather than being membraneous (Figure 3). On the other hand, immunohistochemical analysis of the regional lymph node (medial retropharingeal lymph node) revealed a multifocal strong cytokeratin expression together with a more frequent membranous E-cadherin immunolabeling (Figure 5). Neoplastic cells did not express vimentin within the lymph nodes, but still expressed N-cadherin at nuclear level (Figure 5). Moreover, we detected a slight decrease of the number of neoplastic cells expressing the EMT-related transcription factors (TWIST-1, ZEB-1, and HIF-1α) and nuclear β-catenin (Figure 5 and Figure 6). These findings demonstrate that the morphological changes and the aggressive behavior are most likely due to the EMT process activated within these tumor cells. Considering that the current literature suggests an emerging role for EcPV2 in several equine squamous cell carcinomas [11] and given that a correlation between SSCL and h-HPV infection [6] was demonstrated also in humans, we investigated whether, also in this case, the papillomavirus infection played a role, making the equine species a promising model for this type of tumor.

### 3.3. EcPV2 Detection Suggests a Potential Role in Equine Laryngeal Squamous Cell Carcinomas

DNA obtained from the tumor sample was amplifiable (Table 2) and it showed positivity for EcPV2 L1 (Table 2), with a mean Cq of 22.5 ± 0.17, obtained as mean of three replicates. The negative sample was amplifiable, but negative for EcPV2. Regarding gene expression, only the E6 gene was expressed (Table 3), with a mean Cq of 30.4 ± 0.24.

## 4. Discussion

Based on the current findings, a laryngeal squamous cell carcinoma with a regional lymph node metastasis was diagnosed. The wide immunohistochemical panel used in the current study allowed us to detect a gradual decreased number of cells expressing epithelial markers within the primary tumor site invasive front, together with a gradual increased number of cells expressing mesenchymal markers and key transcription factors for the EMT process. On the other hand, lymph node metastasis revealed a moderate switch in markers expression compared to the primary carcinoma site that might suggest a partial/incomplete mesenchymal to epithelial transition (MET) process. Specifically, lowered numbers of cells expressing E-cadherin and cytokeratin were detected among primary site cancer cells together with aberrant N-cadherin expression and a moderate number of cells immunolabeled for vimentin. These findings were further supported by numerous cells displaying nuclear staining for TWIST-1, ZEB-1, β-catenin, and HIF-1α. In contrast, the metastatic lymph node displayed a partial reversion of the aforementioned epithelial and mesenchymal markers, thus suggesting an MET process. It is well reported that TWIST-1 is considered as the main regulator of EMT [24,25] and is up-regulated in a large number of malignant tumors determining the onset of the metastatic process, via promoting invasiveness in both spontaneous and experimental models [25,26]. Noteworthy, the tumor in the present study had both a high number of cells expressing TWIST-1 and a very low number of cells expressing membranous E-cadherin or, interestingly, the cells exhibited cytoplasmic internalization of the protein. This aberrant cytoplasmic expression has been recently related in equine penile carcinoma to a more aggressive behavior due to AKT/MAPK pathway activation [27,28]. Interestingly, the decreased number of cells expressing E-cadherin (E-cadherin loss) was replaced by the increased number of cells expressing nuclear N-cadherin. The aforementioned results might be in line with a study on human nasopharyngeal carcinoma by Luo and colleagues, reporting a correlation between nuclear N-cadherin and a poorer prognosis [29]. Another aberrant localization found in the current case is represented by β-catenin in both primary and metastatic sites. Normally, the membranous E-cadherin/β-catenin complex maintains the intercellular tight junction and minor free β-catenin cytoplasmic molecules are controlled by multiprotein complexes, while on the other hand, nuclear localization of β-catenin is essential for the progression of various human cancers, such as nasopharyngeal carcinoma, via transcriptional upregulation of downstream genes [30]. These findings are in line with the low number of cells expressing β-catenin at a cytoplasmic level, compared to the higher number of cells expressing β-catenin at a nuclear level found in this study. It is well documented that solid tumors generally have a hypoxic microenvironment [31]. Previous studies suggested that moderate hypoxic conditions might trigger an EMT process via HIF-1α, leading different human cancer cells to significantly increase their invasiveness [32]. Noteworthy, Yang and colleagues demonstrated that HIF-1α directly binds to the hypoxia response element (HRE) in the TWIST promoter, regulating the expression of this transcription factor [33]. These results seem to be in agreement with the HIF-1α expression found in this study. However, all the results regarding the EMT process obtained at a protein level in the present study by immunohistochemistry will need to be further confirmed also at a genetic level in future studies aimed at increasing the knowledge on these aspects by focusing also on the possible role of some microRNA (miRNA) families. The molecular analysis which demonstrated the presence of EcPV2 and its oncogene expression is in agreement with previous studies [11,34,35]. In particular, our data showed E6 but not E2 expression. The lack of E2 expression suggests virus genome integration and loss or disruption of the E2 gene. This can cause deregulation of E6 expression and, in turn, the increase of this event triggers cancer progression [36]. Moreover, a previous study in humans demonstrated a role for E6 in innate immune gene repression [36]. It is also important to consider the potential role of papillomavirus oncoproteins in triggering the EMT process. Interestingly, Liu and colleagues reported that E6 and E7 oncoproteins enhance the expression of HIF-1α, as well as of ZEB-1, SNAIL-1, SLUG, and TWIST-1 in non-small cell lung cancer (NSCLC) cells, thus promoting the EMT process [37]. Recently, a study using human lung samples confirmed the E7 oncoprotein role in promoting EMT in human lung cancers, reporting correlations with E7 and E-cadherin, N-cadherin, and TGF-β expression [38]. According to the authors, the findings of the present work about EcPV oncoproteins and the EMT-related transcription factors and structural/adhesion proteins are in line with the literature and might represent a promising starting point to be further investigated.

## 5. Conclusions

In conclusion, this interesting case of equine metastatic SCCL provides an example of tumor cell adaptation during the metastatic process in the equine species, taking also into account the possible influence of EcPV2 oncoproteins on the EMT process. This is an opportunity to propose the equine species in future studies for evaluation of the potential interactions between EcPV2 oncoproteins and the EMT process both in human and animal cancers, thus opening new study perspectives in this field.

## Figures and Tables

**Table 1:** Details of the antibodies used for immunostaining, including primary antibody, host species, clonality, epitope retrieval method, blocking serum, dilution of primary antibody, secondary antibody, and positive control**.**

Target Antigen	Antibody Details/Clone	Blocking Serum	Heat Induced Epitope Retrieval (HIER)	Primary Antibody Dilution	Secondary Antibody (1:200)	Positive Control
E-cadherin	Monoclonal mouse anti-human, IgG2a, clone 36/E-Cadherin BD 610181 (BD transduction laboratories)	Goat	Microwave 400 W, 3 cycles, 5 min. each, sodium citrate buffer, pH 6.0	1:100	biotinylated goat anti-mouse IgG (BA-1000-Vector Labs)	Horse, skin Dog, skin
Pan-ckytokeratin AE3/AE1	Monoclonal mouse anti-human IgG1 SC-81714 (Santa Cruz Biotechnology)	Goat	Microwave 400 W, 3 cycles, 5 min. each, sodium citrate buffer, pH 6.0	1:100	biotinylated goat anti-mouse IgG (BA-1000-Vector Labs)	Horse, skin Cow, skin
β-catenin	Polyclonal goat anti-human, IgG, AB0095-200 (Sicgen)	Rabbit	Microwave 400 W, 3 cycles, 5 min. each, sodium citrate buffer, pH 6.0	1:3000	biotinylated rabbit anti-goat IgG (BA-1000-Vector Labs)	Horse, intestine Dog, intestine
N-cadherin	Polyclonal rabbit anti-human, IgG, 22018-1-AP (Proteintech)	Goat	Microwave 400 W, 3 cycles, 5 min. each, sodium citrate buffer, pH 6.0	1:3000	biotinylated goat anti-rabbit IgG (BA-1000-Vector Labs)	Horse, heart Cow, heart
Vimentin	Monoclonal mouse anti-human IgG1, Clone V9 (Dako)	Goat	Microwave 400 W, 3 cycles, 5 min. each, sodium citrate buffer, pH 6.0	1:100	biotinylated goat anti-mouse IgG (BA-1000-Vector Labs)	Horse, heart (vessels)
ZEB-1	Polyclonal rabbit anti-human, IgG, LS-C31478 (LSBio)	Goat	Microwave 400 W, 3 cycles, 5 min. each, sodium citrate buffer, pH 6.0	1:200	biotinylated goat anti-rabbit IgG (BA-1000-Vector Labs)	Horse, kidney Dog, kidney
TWIST-1	Polyclonal rabbit anti-human, IgG, Orb329955 (Biorbyt)	Goat	Microwave 400 W, 3 cycles, 5 min. each, sodium citrate buffer, pH 6.0	1:800	biotinylated goat anti-rabbit IgG (BA-1000-Vector Labs)	Horse, kidney
HIF-1α	Polyclonal rabbit anti-human, IgG, NB100-449 (NovusBio)	Goat	Microwave 400 W, 3 cycles, 5 min. each, sodium citrate buffer, pH 6.0	1:1000	biotinylated goat anti-rabbit IgG (BA-1000-Vector Labs)	Horse, kidney Dog, kidney

**Table 2:** Primer set and probes for real-time PCR and RT-real-time PCR.

Gene	Primers	Accession Number	Amplicon (Base Pairs)
Ec-PV2-E2	For: 5’-AAAAGGGAGGGTACGTTGTC-3’ Rev: 5’-CCTGGTAGTAGACATGCTGC-3’	NC_012123.1	90
Ec-PV2-E6	For: 5’-CGTTGGCCTTCTTTGCATCT-3’ Rev: 5’-AGGTTCAGGTCTGCTGTGTT-3’	NC_012123.1	81
Ec-PV2-L1	For: 5’-TTGTCCAGGAGAGGGGTTAG-3’ Rev: 5’-TGCCTTCCTTTTCTTGGTGG-3’	NC_012123.1	81
pEc-PV2-E2	FAM-GCCAAGACAGCCACGACGCCAT-TAMRA	NC_012123.1	22
pEc-PV2-E6	FAM-CCGTGTGGCTATGCTGATGACATTTGG-TAMRA	NC_012123.1	27
pEc-PV2-L1	FAM-CGTCCAGCACCTTCGACCACCA-TAMRA	NC_012123.1	22

**Table 3:** Results of equine papillomavirus type 2 (EcPV2) detection and expression.

Sample ID	Type of Sample	RT-PCR		RT-qPCR	
		B2M	L1	E2	E6
1	Epiglottis tumor	21.1 ± 0.62	26.5 ± 0.17	>40	30.4 ± 0.24
2	Kidney negative sample	21.6 ± 0.27	-	>40	>40

Data are expressed as mean Cq (quantitation cycle) ± SD (standard deviation) of 3 replicates; B2M = beta-2-microglobulin.
